# A study on vertebral refracture and scoliosis after percutaneous kyphoplasty in patients with osteoporotic vertebral compression fractures

**DOI:** 10.1186/s13018-024-04779-9

**Published:** 2024-05-17

**Authors:** Zhichao Qi, Shengli Zhao, Haonan Li, Zhenxing Wen, Bailing Chen

**Affiliations:** 1 Department of Spine Surgery, The First Affiliated Hospital of Sun Yat-sen University, Guangzhou, China; 2 Department of Orthopaedic and Joint Surgery, Qingdao Municipal Hospital, No. 5 Donghai Middle Road, Shinan District, Qingdao City, Shandong Province 266000 P. R. China

**Keywords:** Osteoporotic vertebral compression fracture, Percutaneous kyphoplasty, Scoliosis, Refracture

## Abstract

**Purpose:**

To analyze the association between scoliosis and vertebral refracture after percutaneous kyphoplasty (PKP) in patients with osteoporotic vertebral compression fractures (OVCFs).

**Methods:**

A retrospective study was conducted on 269 patients meeting the criteria from January 2014 to October 2022. All patients underwent PKP with complete data and were followed-up for > 12 months. First, it was verified that scoliosis was a risk factor in 269 patients. Second, patients with scoliosis were grouped based on the Cobb angle to evaluate the impact of the post-operative angle. The cox proportional hazards regression analysis and survival analysis were used to calculate the hazard ratio and recurrence time.

**Results:**

A total of 56 patients had scoliosis, 18 of whom experienced refractures after PKP. The risk factors for vertebral refractures included a T-score < − 3.0 and presence of scoliosis (both *p* < 0.001). The results indicated that the vertebral fractured arc (T10 − L4) was highly influential in scoliosis and vertebral fractures. When scoliotic and initially fractured vertebrae were situated within T10 − L4, the risk factors for vertebral refracture included a postoperative Cobb angle of ≥ 20° (*p* = 0.002) and an increased angle (*p* = 0.001). The mean recurrence times were 17.2 (10.7 − 23.7) months and 17.6 (7.9 − 27.3) months, respectively.

**Conclusion:**

Osteoporosis combined with scoliosis significantly increases the risk of vertebral refractures after PKP in patients with OVCFs. A postoperative Cobb angle of ≥ 20° and an increased angle are significant risk factors for vertebral refractures when scoliotic and initially fractured vertebrae are situated within T10 − L4.

**Supplementary Information:**

The online version contains supplementary material available at 10.1186/s13018-024-04779-9.

## Introduction

Osteoporosis is a systemic bone disease characterized by osteopenia and bone microstructure damage, resulting in increased bone fragility and susceptibility to fractures [1]. The prevalence of osteoporosis is increasing with global population aging, leading to a substantial economic burden [2]. Osteoporotic vertebral compression fractures (OVCFs) are the most serious consequence of osteoporosis, leading to intractable back pain and an increased risk of complications and mortality [3, 4]. Percutaneous kyphoplasty (PKP), a minimally invasive method for treating OVCFs, provides clear short- and long-term benefits for patients by promptly relieving back pain and restoring the height of collapsed vertebrae [5–7].

Although the advantages of PKP have been well demonstrated, vertebral refractures after PKP have been reported at rates ranging from 10 to 29.4%, affecting both adjacent and distant vertebrae [8–10]. There are many risk factors for refractures after PKP [11], and sagittal spinal imbalance has been acknowledged as the predominant contributor in previous studies [8, 12–14]. In our prior research, however, we observed a high prevalence of osteoporosis among patients aged > 50 years who underwent spinal surgery, especially among those primarily diagnosed with degenerative scoliosis [15]. Adult degenerative scoliosis is a complex condition with a multifactorial etiology. Osteoporosis contributes to degenerative scoliosis by reducing bone density and increasing bone fragility [16]. These findings imply that coronal stability may be habitually overlooked.

Fang et al. [17] found that combined scoliosis was an independent risk factor for vertebral refracture after PKP. However, they did not explicitly evaluate the association between the scoliotic vertebrae and the fractured vertebrae. There are also few satisfactory descriptions of the position of postoperative vertebral refracture. Therefore, the present study aimed to further analyze the association between scoliosis and vertebral refracture after PKP in patients with OVCFs.

## Methods

### Study subjects

This single-center retrospective study was approved by the institutional review board and ethics committee of the research institution and was supported by the clinical research center (No [2021] 020).

The patients were diagnosed with OVCFs and underwent PKP at The First Affiliated Hospital of Sun Yat-sen University from January 2014 to October 2022. The diagnosis was based on physical examination and radiographic results. The patients’ chief complaints were back pain and its impact on their daily activities before the initial surgery.

**Fig. 1:**
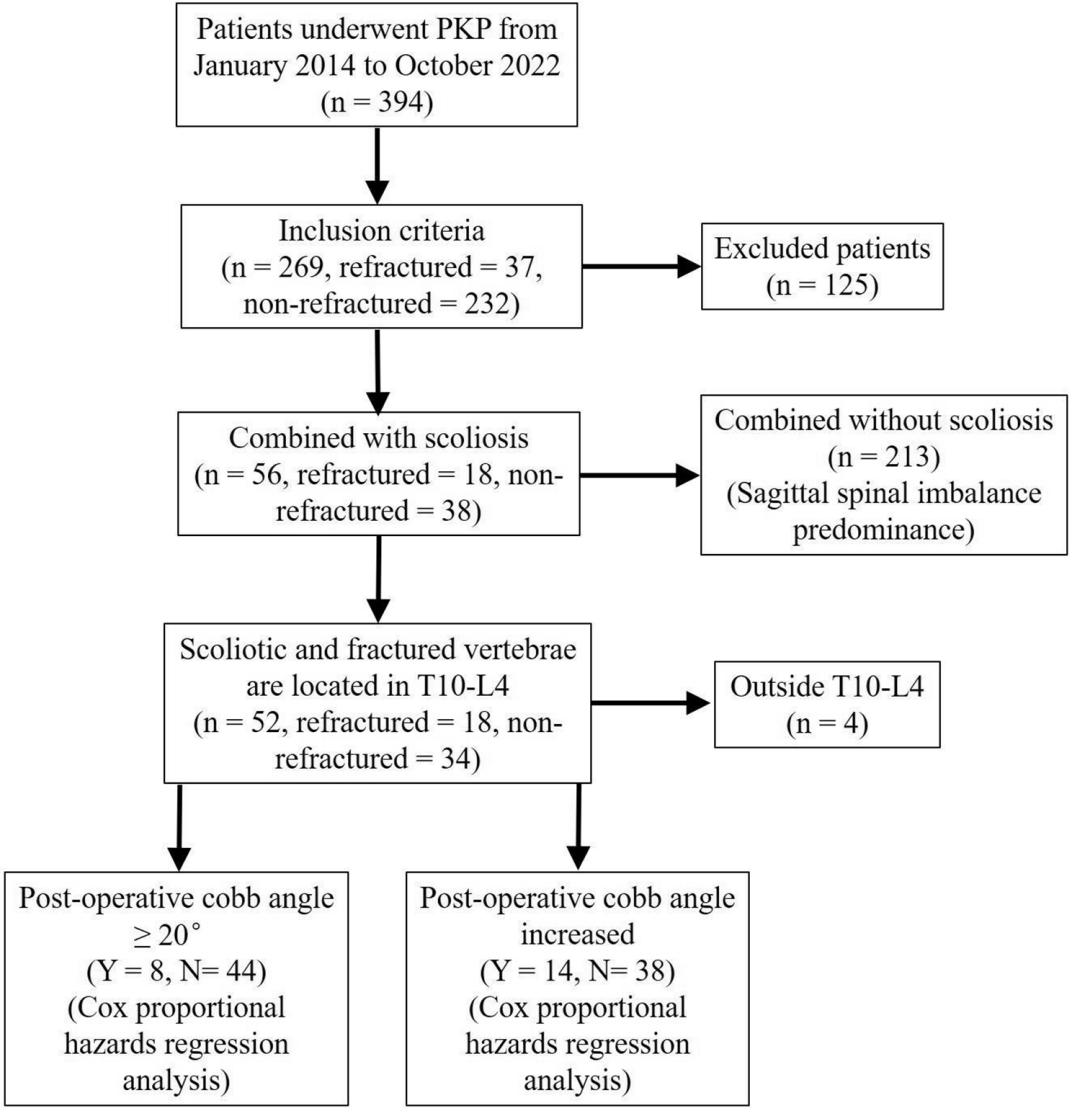
Flowchart of patient recruitment

The inclusion criteria were (1) primary non-traumatic OVCFs that occurred spontaneously without explicit violent forces; (2) age ≥ 50 years; (3) underwent PKP. The exclusion criteria were (1) history of spinal surgery or back soft tissue surgery; (2) vertebral fracture due to spinal tumor, vertebral metastatic tumor, tuberculosis of the spine, inflection, ankylosing spondylitis, or connective tissue diseases affecting the bone; (3) history of violent trauma; (4) fracture in the posterior vertebral column; and (5) inability to stand or complete radiography due to physical limitations or pain. The patients’ classification during the research process is detailed in the flowchart (Fig. 1).

### Data collection

The patients were routinely examined with plain radiographs to diagnose and assess OVCFs. Anteroposterior and lateral radiographs were routinely taken in the standing position one day before and one day after surgery. The patients were required to complete the Visual Analogue Scale (VAS) before and after surgery once they undergone radiography. During follow-up, the patients were required to undergo anteroposterior and lateral spinal radiographs in a standing position under two specific conditions: when they experienced the same pain and discomfort as before and when they returned to the hospital for postoperative follow-up on schedule. Only the first refracture was included in the analysis for patients who experienced multiple recurrences during the follow-up period. The patients were followed up for at least 12 months.

Patient hospitalization information, including sex (male/female), age (years), follow-up time (months), body mass index (BMI; kg/m^2^), bone mineral density (BMD; T-score), multi-segment vertebral fractures (Y/N), hypertension (Y/N), diabetes mellitus (Y/N), fall history (Y/N), length of hospital stay (days), operative time (min), blood loss volume (mL), bone cement volume (mL), preoperative VAS score, postoperative VAS score, and presence of scoliosis (Y/N), was collected.

The data and files were reviewed and collected by one researcher and measured and analyzed by another researcher in a single-blinded manner.

### Radiographic parameters

Thoracic kyphosis (TK) was determined from the angle between the upper endplate of the fourth thoracic vertebra (T4) and the inferior endplate of T12, and lumbar lordosis (LL) was determined from the angle between the upper endplate of the first lumbar vertebra (L1) and the inferior endplate of L5. Furthermore, pelvic tilt (PT) was determined from the angle between a vertical line passing through the center of the femoral head and a line joining this point and the midpoint of the sacral endplate, and sacral slope (SS) was determined from the angle between the horizontal line and the superior endplate of S1. Pelvic incidence (PI) refers to the angle between the perpendicular line passing through the midpoint of the sacral endplate and a line joining this point and the center of the femoral head. Sagittal vertical axis (SVA) was defined as the sagittal offset of a plumb line dropped from the center of the C7 vertebral body to the posterosuperior corner of the sacral endplate. Sagittal imbalance was defined as a case in which the SVA was ≥ 5 cm and the PT was ≥ 20° [18]. The cobb angle was measured on anteroposterior radiographs in the coronal plane, and a Cobb angle exceeding 10° was defined as scoliosis preoperatively or postoperatively [19].

### BMD evaluation

The BMD (T-score) was measured at the lumbar spine (L1–L4), total hip, and femoral neck using the Lunar iDXA (GE Healthcare, Chicago, IL, US). A single device was used for the whole study. Fractured vertebrae were excluded from the measurement. A T-score of ≤–2.5 at the femoral neck was used to define osteoporosis, and a T-score of <–3 was defined as severe osteoporosis.

### Statistical analysis

SPSS 25.0 statistical software (SPSS Inc., Chicago, IL, US) was used for data processing. The image measurement data were expressed as the mean ± standard deviation (x ± s). Two independent-samples t-tests were used to compare the different groups. The chi-square test was used to compare categorical variable data. To determine whether scoliosis affected refractures in terms of the occurrence time, the log-rank test was performed using Kaplan-Meier survivorship analysis. The Cox proportional hazards model was used to analyze each risk factor. The multivariate analysis was performed on variables with p-values of < 0.1 in the univariate analysis to assess risk factors. A p-value of < 0.05 was considered statistically significant.

## Results

### Baseline characteristics

After excluding patients with incomplete hospitalization information, a total of 269 patients met the inclusion and exclusion criteria. Thirty-seven patients (13.8%) demonstrated refracture after PKP during the follow-up period, and the ratio of males to females was 4:33. A total of 56 patients had scoliosis, 18 of whom experienced refractures after PKP.

Statistically significant differences were detected between the refractured group and the non-refractured group in terms of BMD, severe osteoporosis, multi-segment vertebral fractures, TK, SVA, SVA ≥ 5 cm, and combined scoliosis (all *p* < 0.05). No statistically significant differences were detected between the two groups in terms of sex, age, age > 70 years, follow-up time, BMI, hypertension status, diabetes status, fall history, hospital stay, operative time, blood loss, bone cement, preoperative VAS score, postoperative VAS score, SS, PT, PI, LL, and sagittal imbalance (all *p* > 0.05) (Table 1).

**Table 1:** Demographic data

Characteristics	Total (*N* = 269)	Refractured (*N* = 37)	Non-refractured (*N* = 232)	*p*-value
Sex (M/F)	50/219	4/33	46/186	0.190
Age (years)	73.0 ± 8.3	74.7 ± 8.4	72.7 ± 8.3	0.179
Age > 70y	166 (61.7%)	25 (67.6%)	144 (60.8%)	0.430
Follow-up (months)	24 ± 14.7	22.5 ± 16.6	24.3 ± 14.3	0.481
BMI (Kg/m^2^)	23.4 ± 3.0	23.5 ± 2.8	23.4 ± 3.0	0.858
BMD (T-score)	-3.1 ± 0.8	-3.8 ± 0.7	-3.0 ± 0.8	< 0.001
Severe osteoporosis	136 (50.6%)	31 (83.8%)	105 (45.3%)	< 0.001
Multi-segment	91 (33.8%)	21 (56.8%)	70 (30.2%)	0.002
Hypertension	72 (26.8%)	9 (24.3%)	63 (27.2%)	0.718
Diabetes	48 (17.8%)	8 (21.6%)	40 (17.2%)	0.518
Fall history	194 (72.1%)	27 (73.0%)	167 (72.0%)	0.901
Hospital stays (days)	4.2 ± 1.6	4.5 ± 1.8	4.2 ± 1.6	0.240
Operative time (min)	46.8 ± 14.4	49.5 ± 18.8	46.3 ± 13.5	0.335
Blood loss (mL)	3.4 ± 2.0	3.7 ± 1.6	3.3 ± 2.1	0.350
Bone cement (mL)	3.4 ± 0.9	3.3 ± 1.0	3.4 ± 0.9	0.722
Preoperative VAS	7.3 ± 0.6	7.5 ± 0.6	7.3 ± 0.6	0.072
Postoperative VAS	3.3 ± 0.9	3.2 ± 0.8	3.3 ± 0.9	0.876
SS	35.5 ± 8.8	33.7 ± 8.6	35.8 ± 8.8	0.161
PT	18.3 ± 6.3	19.7 ± 7.0	18.1 ± 6.3	0.147
PI	53.8 ± 10.0	53.4 ± 11.3	53.9 ± 9.8	0.755
LL	28.4 ± 13.5	30.8 ± 13.2	28.1 ± 13.5	0.244
TK	32.3 ± 15.1	39.0 ± 17.8	31.2 ± 14.3	0.003
SVA	3.9 ± 2.4	4.8 ± 3.0	3.8 ± 2.3	0.020
SVA ≥ 5 cm	68 (25.3%)	15 (40.5%)	53 (22.8%)	0.021
Sagittal imbalance	35 (13%)	6 (16.2%)	29 (12.5%)	0.597
Scoliosis	56 (20.8%)	18 (48.6%)	38 (16.4%)	< 0.001

BMI, body mass index; BMD, bone mineral density; VAS, visual analogue scale; SS, sacral slope; PT pelvic tilt; PI pelvic incidence; LL lumbar lordosis; TK thoracic kyphosis; SAV sagittal vertical axis

### Survival analysis

In the univariate analysis, severe osteoporosis, multi-segment vertebral fractures, SVA ≥ 5 cm, and presence of scoliosis were risk factors for refracture after PKP (Table 2). In the multivariate analysis, presence of scoliosis (hazard ratio [HR] 0.234, 95% confidence interval [CI] 0.116–0.474, *p* < 0.001) and severe osteoporosis (HR 0.156, 95% CI 0.063–0.387, *p* < 0.001) were risk factors for refracture after PKP.

**Table 2:** Risk factors for refracture in the univariate analysis

Risk factors	B	Standard error	Wald	*p*-value	Exp (B)	95% CI
Sex	0.593	0.530	1.252	0.261	1.809	0.640—5.114
Age > 70	-0.328	0.352	0.866	0.352	0.721	0.362—1.437
Severe osteoporosis	-0.946	0.225	17.727	< 0.001	0.388	0.250—0.603
Multi-segment	-0.549	0.170	10.476	0.001	0.578	0.414—0.805
SVA ≥ 5 cm	-0.489	0.174	7.884	0.005	0.613	0.436—0.863
Sagittal imbalance	-0.218	0.226	0.927	0.336	0.804	0.516—1.253
Scoliosis	-0.766	0.170	20.325	< 0.001	0.465	0.333—0.648

SAV sagittal vertical axis

**Fig. 2:**
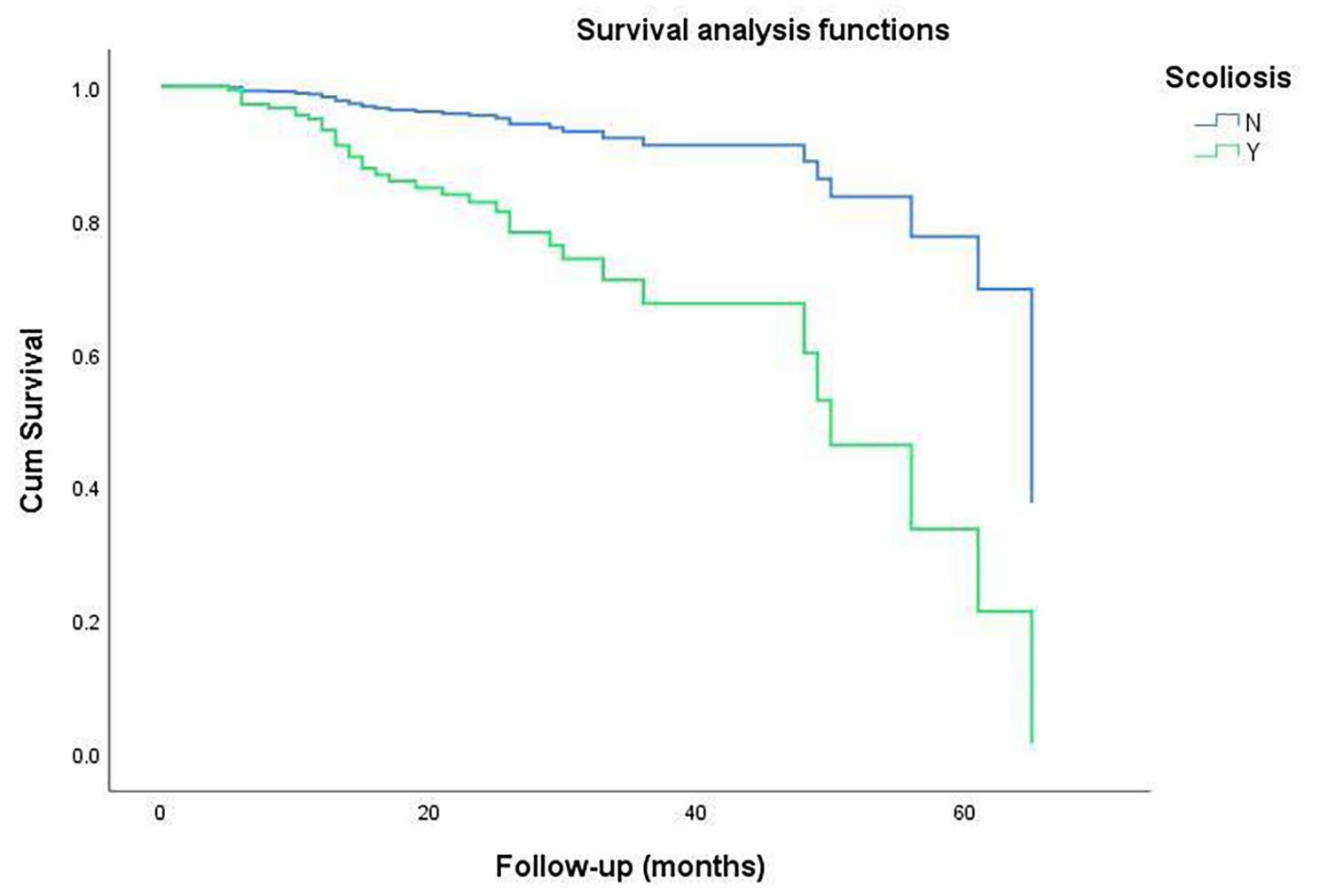
The survival analysis suggested that OVCFs combined with scoliosis were associated with the occurrence of vertebral refracture

The survival analysis revealed that the mean refracture duration was 39.5 (33.1–45.9) months in the scoliotic group and 61.7 (57.0–66.4) months in the non-scoliotic group (*p* < 0.001; Fig. 2).

### Scoliosis and vertebral fracture

Upon counting and sorting the fractured and scoliotic vertebrae, it was observed that 85.4% of all scoliotic vertebrae in the non-refractured group and 93.6% in the refractured group spanned from T10 to L4. In the non-refractured group, 96.8% of initially fractured vertebrae were located in the T10–L4 region. Initially fractured vertebrae (90.3%) and refractured vertebrae (88.9%) were predominantly located in the T10–L4 region within the refractured group.

**Fig. 3:**
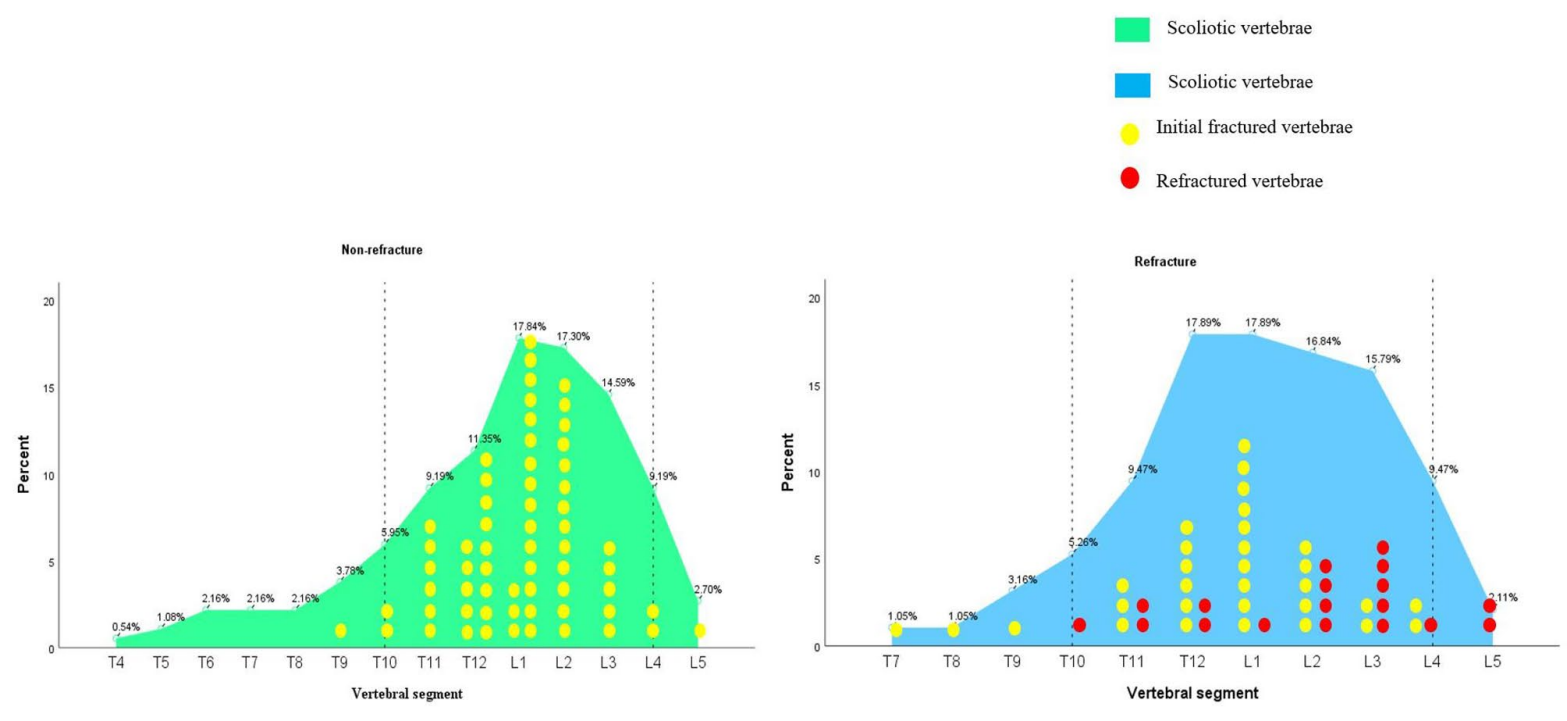
Distribution of scoliotic and fractured vertebrae in the refractured and non-refractured groups

This area should be emphasized as a high-incidence region for vertebral fractures and refractures combined with scoliosis, which may be referred to as the vertebral fractured arc (Fig. 3). In this area, initially fractured vertebrae and refractured vertebrae are also part of scoliotic vertebrae.

With the exception of four patients whose initial fractured vertebrae or scoliotic vertebrae were outside of the vertebral fractured arc, the mean BMD of the refractured group (–3.7 ± 0.9) was lower than that of the non-refractured group (–3.2 ± 0.7) (*p* < 0.05). The mean postoperative Cobb angle (*p* < 0.001) and variational Cobb angle (*p* < 0.05) in the refractured group were significantly greater than those in the non-refractured group. The percentage of patients with a postoperative Cobb angle of ≥ 20° was 44.4%, and the proportion was significantly greater in the refractured group than in the non-refractured group (*p* < 0.001) (Table 3).

**Table 3:** Demographic data of patients with scoliosis

	Refractured (*N* = 18)	Non-refractured (*N* = 34)	*p*-value
Sex (M/F)	3/15	6/28	0.929
Age (years)	75.9 ± 8.5	73.5 ± 8.2	0.334
BMI (Kg/m^2^)	23.1 ± 3.0	23.5 ± 3.3	0.675
BMD (T-score)	-3.7 ± 0.9	-3.2 ± 0.7	0.014
Severe osteoporosis	14(77.8%)	18(52.9%)	0.080
Preoperative VAS	7.4 ± 0.6	7.3 ± 0.6	0.413
Postoperative VAS	3.5 ± 0.7	3.5 ± 0.9	0.907
Cobb angle (°)			
-Preoperative	18.3 ± 10.1	15.4 ± 3.9	0.249
-Postoperative	19.0 ± 6.3	11.6 ± 2.8	< 0.001
-Variation	0.7 ± 5.7	-3.8 ± 3.9	0.006
-Post-op ≥ 20°	8 (44.4%)	0 (0%)	< 0.001
-Post-op increased	10 (55.6%)	4 (11.8%)	0.002
Follow-up (months)	18.2 ± 14.1	23.2 ± 12.5	0.194

BMI, body mass index; BMD, bone mineral density; VAS, visual analogue scale

In the univariate analysis, the risk factors for refracture included severe osteoporosis (HR 0.410, 95% CI 0.195–0.863, *p* = 0.019), an increased postoperative Cobb angle (HR 0.371, 95% CI 0.222–0.621, *p* < 0.001) and a postoperative Cobb angle of ≥ 20° (HR 0.396, 95% CI 0.244–0.642, *p* < 0.001).

The multivariate analysis revealed that the risk factors for refracture included an increased postoperative Cobb angle (HR 0.178, 95% CI 0.062–0.511, *p* = 0.001) and a postoperative Cobb angle of ≥ 20° (HR 0.197, 95% CI 0.071–0.548, *p* = 0.002).

**Fig. 4:**
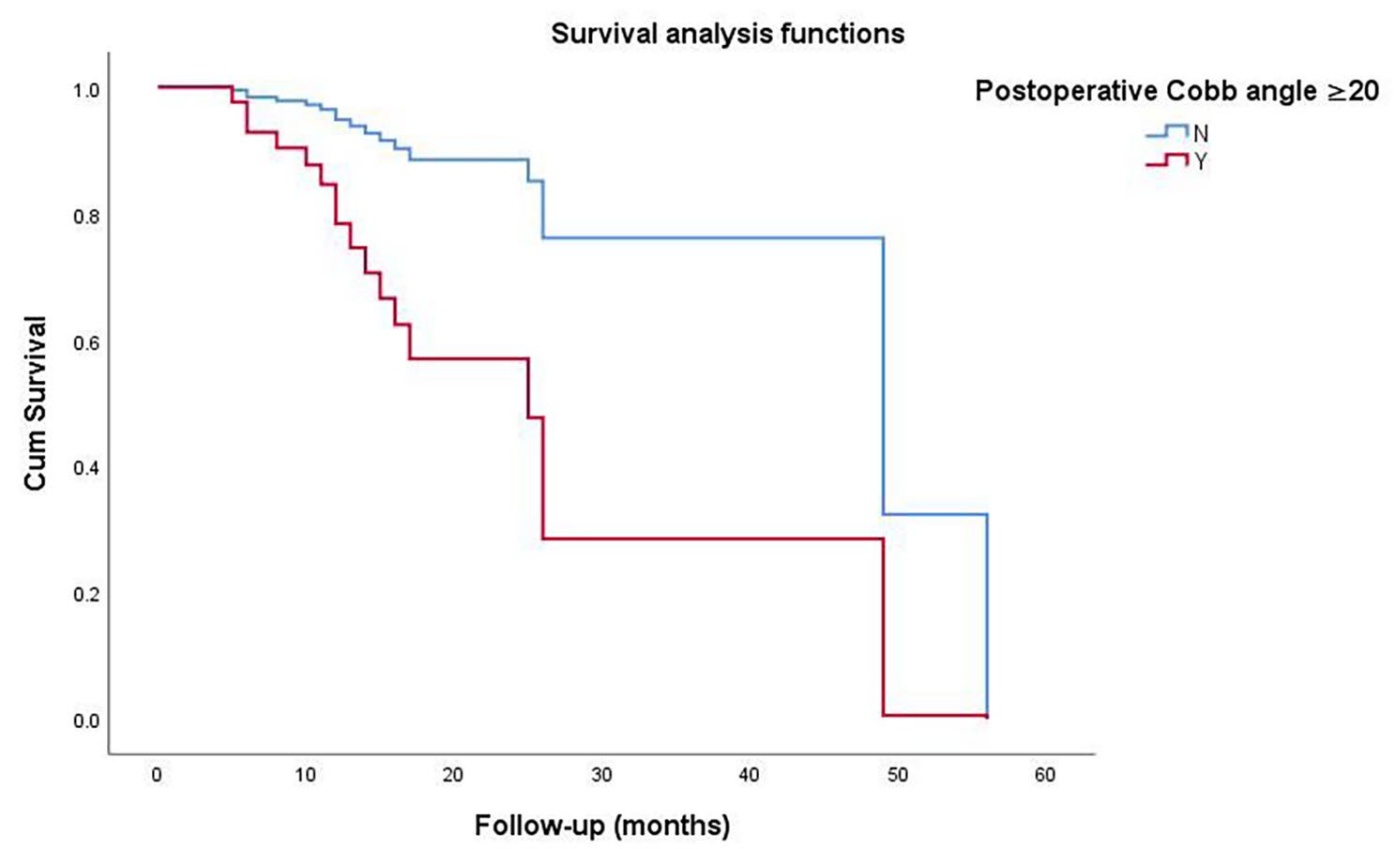
The survival analysis suggested that a postoperative Cobb angle of ≥ 20° was associated with the occurrence of vertebral refracture combined with scoliosis

**Fig. 5:**
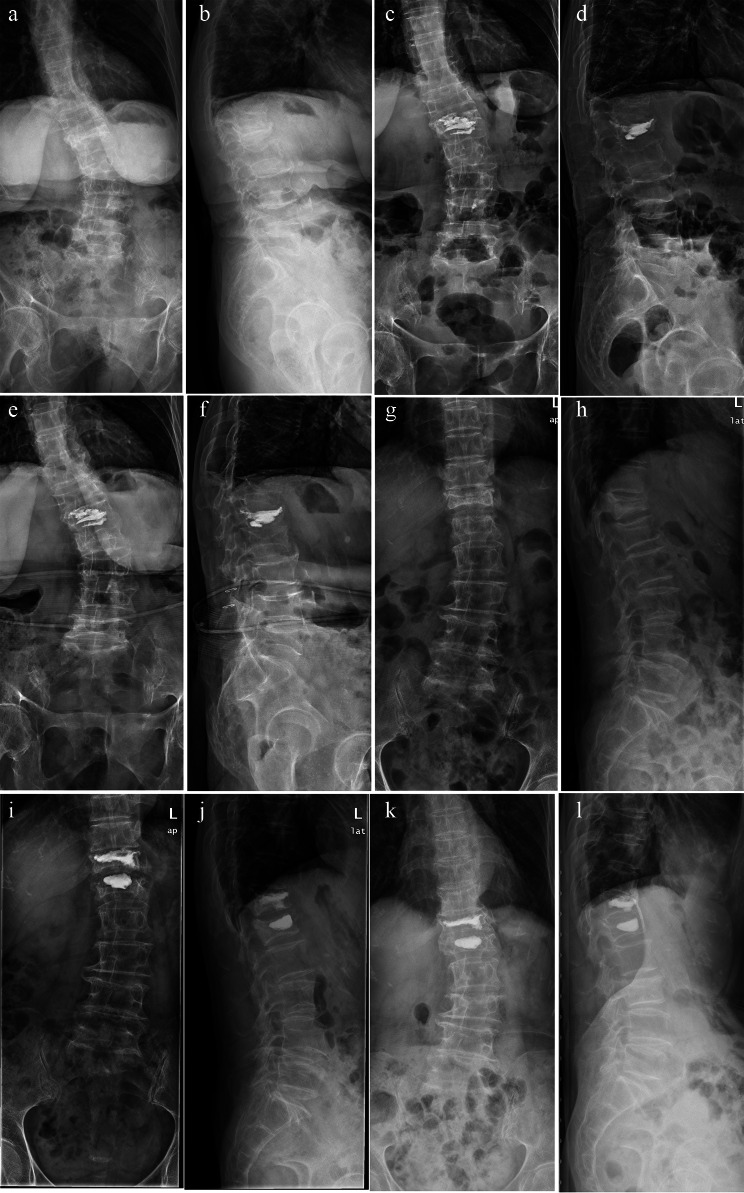
**a**–**f**. A 65-year-old female patient with a primary diagnosis of L1 OVCF (2020-12-14) and degenerative scoliosis (T12–L4). **a**, **b**. The X-ray film showed L1 vertebral fracture and degenerative scoliosis (Cobb angle of 24°) before surgery. **c**, **d**. The X-ray film showed that the Cobb angle was 21° one day after PKP. Although the postoperative angle had decreased slightly, it was still greater than 20°. **e**, **f**. The X-ray film showed a refracture of the L2 vertebra and a Cobb angle of 27° for scoliosis after 14 months. g–l. A 69-year-old female patient with a primary diagnosis of T12 and L1 OVCF (2016-8-22) and degenerative scoliosis (T12–L5). 5 **g**, **h**. The X-ray film showed that Cobb angle was 16° before surgery. 5 **i**, **j**. The X-ray film showed that the Cobb angle was 19° one day after PKP. 5 **k**, **l**. The X-ray film showed a refracture of the L3 vertebra and a Cobb angle of 21° for scoliosis after 17 months

The mean recurrence time for patients with a postoperative Cobb angle of ≥ 20° was 17.6 (7.9–27.3) months. The mean recurrence time for patients with an increased postoperative Cobb angle was 17.2 (10.7–23.7) months. The survival analysis verified that these two conditions significantly increased the risk of vertebral refracture (*p* < 0.001; Fig. 4; Table 4). Typical cases are shown in Fig. 5.

**Table 4:** Different recurrence time after PKP

**a. Differences between postoperative Cobb angle < 20° and ≥ 20°**			
	**Survival period (months)**		*p*-value
	**Cobb angle < 20°**	**Cobb angle ≥ 20°**	
Mean (95%CI)	44.2 (37.0—51.3)	17.6 (7.9—27.3)	<0.001
Median (95%CI)	56.0	14.0 (9.1—18.9)	
**b. Differences between postoperative Cobb angle increasing and Cobb angle reduced or unchanged**			
	**Survival period (months)**		*p*-value
	**Angle increased**	**Angle reduced or unchanged**	
Mean (95%CI)	17.2 (10.7—23.7)	45.7 (38.7—52.8)	<0.001
Median (95%CI)	15.0 (10.9—19.1)	56.0	

## Discussion

In this study, a total of 269 patients diagnosed with primary osteoporosis underwent PKP and were followed up for > 12 months to assess the occurrence of new vertebral fractures. Thirty-seven patients (13.8%) experienced vertebral refracture. We compared the data between the refractured group and the non-refractured group to determine the significant risk factors identified previously [11, 14, 17]. Bone cement leakage was not observed in 269 patients and was not analyzed in this study. The Cox regression analysis confirmed that severe osteoporosis and scoliosis significantly increased the risk of vertebral refractures after PKP in patients with OVCFs.

Moreover, an SVA of ≥ 5 cm was identified as a risk factor in the univariate analysis, but it did not emerge as a risk factor in the multivariate analysis in this sample of 269 patients. After excluding the 56 patients with scoliosis, we analyzed the data of the remaining 213 patients. In the 213 patients, an SVA of ≥ 5 cm was a significant risk factor in both the univariate analysis (HR 0.537, 95% CI 0.332–0.871, *p* = 0.012) and the multivariate analysis (HR 0.370, 95% CI 0.140–0.980, *p* = 0.045). The comparison of these results emphasized that the presence of scoliosis was a risk factor for the refracture of OVCFs after PKP, which is different from sagittal instability. These results are consistent with previous findings [17].

The sagittal balance of the spine plays an important role in maintaining normal biomechanical and physiological functions [18, 20]. Baek et al. [21] reported that sagittal parameters, including SVA ≥ 6 cm, segment kyphotic angle ≥ 11°, SS < 25°, and LL < 25°, were significantly associated with mono-segment adjacent vertebral refracture after percutaneous vertebroplasty. Moreover, the forward tilt of the upper part of the body resulting from an OVCF cannot be corrected completely through vertebroplasty [22]. In this study, although spinal sagittal instability may not be the dominant risk factor for OVCFs in patients with scoliosis, it still played a pivotal role in OVCFs in patients without scoliosis. The importance of spinal sagittal stability has been widely recognized, and PKP has a positive effect on the correction of spinal sagittal instability [23]. In addition, rehabilitation management and anti-osteoporotic treatment are capable of increasing the quality of life of patients and reducing the occurrence of vertebral refractures [24, 25]. We routinely provided patients with postoperative health education and believe that postoperative health education for patients and their families was an important part of treatment because the patients paid particular attention to their posture and spinal function in their daily lives.

By analyzing the association between scoliotic vertebrae and fractured vertebrae, we detected a high-frequency intersection area between the two. This area contained vertebrae from T10 to L4, and we termed this the vertebral fractured arc. Scoliotic vertebrae (85.4%) and initial fractured vertebrae (96.3%) located within the vertebral fractured arc were observed in non-refractured scoliotic patients. Scoliotic vertebrae (93.6%), initially fractured vertebrae (90.3%), and refractured vertebrae (88.9%) were located within the vertebral fractured arc in refractured scoliotic patients. OVCFs are widely acknowledged to primarily occur in the thoracolumbar (T10–L2) region, with secondary occurrences in the L3–L5 vertebrae [26]. The thoracolumbar region connects the thorax to the lumbosacral region with a large range of activity and experiences a large stress change, which is associated with a high incidence of stress fracture. Degenerative scoliosis resulting from osteoporosis typically occurs in the lumbar and thoracolumbar junction, accompanied by intervertebral disc degeneration and vertebral deformation [27]. According to the study results, we concluded that a slightly “S”-shaped vertebral fractured arc should be applicably used for studying OVCFs combined with scoliosis.

Four patients were excluded because they had scoliotic or fractured vertebrae without the vertebral fractured arc. These four patients did not demonstrate vertebral refracture during the follow-up period, and their postoperative Cobb angle varied by 0°. A postoperative Cobb angle of ≥ 20° and an increased postoperative Cobb angle were identified as risk factors for vertebral refracture in the analysis of 52 patients, both in the univariate and multivariate analyses. The recurrence time significantly increased in both conditions. Interestingly, we observed that all of the refractured vertebrae and almost all of the initial fractured vertebrae were situated within the scoliotic curve. James et al. [28] reported that the curve of degenerative scoliosis involved the area from T12 to L5 with the apex at L2 or L3. Approximately 73% of patients had an annual progression of 3° within 5 years. Degenerative scoliosis is more prevalent in females than in males because postmenopausal osteoporosis increases vertebral bone fragility and contributes to disease progression [29]. Although the scoliotic vertebral BMD is mildly higher than the hip BMD within the same patient, the rate of decrease in the vertebral BMD of patients with scoliosis with age is more significant than that of patients without scoliosis [30]. Furthermore, scoliosis causes uneven force on the vertebrae, leading to significant differences in calcium balance and bone strength [31].

Abnormal spinal structure may result in inhomogeneous stress on the vertebrae. We inferred that cortical bone may maintain relative strength and shape, but cancellous bone could be hollowed out owing to the transfer of calcium and minerals from cancellous bone to cortical bone as a result of stress-induced abnormal bone metabolism. The unstable vertebral structure made it easier to create multiple opportunities for fractures, and osteoporosis and scoliosis exacerbate each other.

Anti-osteoporosis treatment is undoubtedly the first step in preventing vertebral refractures. Based on our study results, we recommend controlling coronal scoliosis to resist natural growth of the curvature and maintaining the postoperative Cobb angle at ≤ 20° when treating OVCFs combined with degenerative scoliosis in the vertebral fractured arc. If the patient’s BMD is <–3 T simultaneously, it should be considered as a severe OVCFs. In these patients, simple PKP surgery may no longer be effective. Zhou et al. [32] suggested using posterior pedicle screw fixation + PKP to maintain spinal sagittal balance and achieve better long-term clinical outcomes in elderly patients with severe OVCFs. Our previous study also demonstrated that short-segment fixation with cement-reinforced screws rather than long-segment fixation was the most satisfactory procedure for treating severe OVCFs [33].

This study has several limitations. First, this was a retrospective study, which may have caused inherent bias in the data and incomplete data collection. However, the study relied heavily on objective imaging parameters, which were less affected. Low patient compliance with postoperative follow-up may also have caused incomplete data. Some patients become accustomed to avoiding seeking medical attention unless absolutely necessary, which means that some patients with refractures may have been neglected. Nevertheless, we used the Kaplan-Meier survival analysis to minimize deviation. Second, new fractures only included those with symptoms that occurred during the follow-up period or fractures that were identified by radiographic examination during the planned period. Therefore, asymptomatic fractures may not have been collected, and cases of multiple refractures were excluded. Third, postoperative radiographic images did not include the total spine bending position, making it impossible to estimate the spinal flexibility for analyzing scoliosis. Finally, although we recommend that patients diagnosed with osteoporosis continue with anti-osteoporosis treatment, some patients may not follow medical advice for economic and other reasons.

## Conclusion

Our research demonstrated that osteoporosis combined with scoliosis significantly increases the risk of vertebral refractures after PKP in patients with OVCFs. In particular, when the scoliotic vertebrae and initially fractured vertebrae were located within the vertebral fractured arc (T10–L4), a postoperative Cobb angle of ≥ 20° and an increased Cobb angle were significant risk factors for vertebral refractures.

### Electronic supplementary material

Below is the link to the electronic supplementary material.

Supplementary Material 1

Supplementary Material 2

## Data Availability

No datasets were generated or analysed during the current study.
